# Exploring dance movement therapy as a novel approach to improving heart rate variability in healthy older people

**DOI:** 10.3389/fpsyg.2026.1810072

**Published:** 2026-10-07

**Authors:** Laura Sebastiani, Said Daoudagh, Giacomo Ignesti, Marina Raglianti, Paolo Paradisi

**Affiliations:** 1 Department of Translational Research on New Technology in Medicine and Surgery, University of Pisa, Pisa, Italy; 2 Institute of Information Science and Technologies “A. Faedo”, ISTI-CNR, Pisa, Italy; 3 Departamento de Educación de la Facultad de Educación y Humanidades, Universidad de Tarapacá, Arica, Chile; 4 BCAM-Basque Center for Applied Mathematics, Bilbao, Spain

**Keywords:** active aging, cardiac function, dance movement therapy, heart rate variability, signal processing, wellbeing

## Abstract

A decline in cardiac autonomic control, as assessed by heart rate variability (HRV), is considered a natural component of aging. However, physical exercise, including dance-based activities, can be effective in preventing/reversing this decline. Aim of this study was to verify whether Dance Movement Therapy (DMT), which uses dance, movement, body awareness and embodied interpersonal communication to promote wellbeing, improves vagally-mediated HRV, which has been linked to physical and mental health, in a sample of people aged over 65. To this purpose, HRV-derived vagal and sympathetic indices, obtained by electrocardiogram signals, as well as tonic skin conductance level, which is a marker of tonic sympathetic activity, recorded during two rest sessions that preceded and followed a 8-week DMT activity, were compared through repeated measures analysis of variance. Results showed post-DMT changes in HRV-derived vagal parameters (root mean square of the successive differences between RR intervals (RMSSD), standard deviation of the beat-to-beat changes in RR intervals (short-term HRV) (SD1) and high frequency power (HF)) in the time, frequency and nonlinear domains. More specifically, the root mean square of the successive differences between RR intervals (RMSSD), the high frequency power (HF) and the standard deviation of the beat-to-beat changes in RR intervals (short-term HRV) (SD1) were higher in the post- than pre-DMT session. Conversely, no changes were found in HRV sympathetic indices nor in skin conductance level. The post-DMT improvement in vagally-mediated HRV in healthy old people is a new finding and we argue that this beneficial effect may be due to DMT's ability to enhance social interaction and promote wellbeing.

## 1 Introduction

Heart rate variability (HRV), which is defined as the variation in the time intervals between successive heartbeats (RR), is widely used by the scientific community to indirectly investigate the dynamics of the autonomic system based on electrocardiogram measurements (Laborde et al., 2017; Shaffer and Ginsberg, 2017).

The study of HRV employs a variety of non-invasive metrics, including statistical indices that span from time averages and frequency-based analyses to nonlinear, or complex, indices, which reflect the continuous and dynamic interplay between two branches of the autonomic nervous system: the sympathetic nervous system (SNS), which prepares the body for action, and the parasympathetic nervous system (PSNS), which promotes rest and recovery (Shaffer and Ginsberg, 2017). A high HRV is associated with particularly strong PSNS activity, which is mediated by the vagus nerve, and indicates good recovery, general physical fitness and well-balanced autonomic control. Conversely, low HRV reflects increased SNS activity and/or vagal withdrawal, and is indicative of the body's response to stress, illness or overtraining (Laborde et al., 2017).

HRV has also proved useful for investigating mental health. Actually, enhanced cognitive functions have been linked to high, vagally mediated, HRV at rest, such as mental flexibility, working memory, attention regulation, motor imagery (Colzato and Steenbergen, 2017; Sebastiani et al., 2019; Stenfors et al., 2016). In contrast, low resting state HRV values have been linked to cardiovascular disturbances, as well as to impaired adaptability to both internal and external stimuli (Ernst, 2017).

Within the context of aging, a reduction in HRV indices (e.g., RMSSD, HF, and LF power) is widely accepted as a natural physiological process (De Meersman and Stein, 2007). Lower cardiac vagal control in older adults is often associated with cognitive impairment and functional decline (Arakaki et al., 2023). However, many studies on age-related HRV changes have focused primarily on clinical populations. For instance, older adults with depression show lower LF-HRV, reflecting reduced baroreflex function (Brown et al., 2018), along with a progressive increase in sympathetic tone over time (Rouch et al., 2022). It is interesting to note that studies conducted on cohorts of healthy individuals have not observed any age-related decline in HRV in older adults with minimal physical or psychological symptoms (Tan et al., 2019) and have also shown that the parasympathetic indices of HRV decline rapidly up to the fourth decade of life, before remaining relatively stable thereafter in healthy individuals. Therefore, when assessing alterations of cardiac autonomic control in older individuals, other factors besides old age must be taken into consideration. For instance, evidence has been presented which demonstrate that lifestyle factors, such as physical activity levels, can influence the body's normal functioning and modulate HRV (Gässler et al., 2021). Indeed, the tendency toward sedentary behavior that is frequently exhibited by the older population has been linked to an elevated prevalence of cardiovascular diseases (Archer and Blair, 2011). Conversely, the efficacy of aerobic exercise in enhancing metabolic and cardiovascular health in older adults has been demonstrated (Cadore et al., 2014), thereby indicating that effective strategies can be employed to prevent or reverse HRV deterioration in this demographic. In particular, dancing represents a form of aerobic exercise with which older adults can easily engage, and research has demonstrated that dancing can yield a number of benefits for this demographic (Esmail et al., 2020).

The physical advantages obtained were comparable to those achieved through physical exercise training, including improvements in flexibility and postural stability (Hwang and Braun, 2015). A positive impact has been observed in the domain of psychosocial wellbeing and in sleep quality. (Kaltsatou et al., 2014; Song et al., 2024). An exergaming-based dance training protocol was recently shown to improve heart rate variability (HRV) in healthy older adults (Varas-Diaz et al., 2020). Following a six week training, participants showed an improvement in HF power, RMSSD and pNN50 compared to the control group.

In this regard, Dance Movement Therapy (DMT) is a particularly noteworthy approach as it emphasizes the integration of emotional, physical and social dimensions. In fact, DMT^1^ is a complementary therapy that promotes emotional, cognitive, physical and social integration through interactions between individuals mediated by body movements (Winters Fisher, 2019; Dulicai and Hill, 2007). DMT emphasizes the expressive and qualitative dimensions of movement as vehicles for improving psychological and emotional wellbeing (Goodill, 2025) and is based on the assumption of a dynamic relationship between mind, body and social interactions, which includes both affective and cognitive processes. It is rooted in the theoretical framework of *embodied cognition*, which posits that cognitive processes are intrinsically shaped and modulated by our body's engagement with the surrounding environment, including the social environment (de la Parra López and Panhofer, 2023; Goodill, 2025; Paradisi et al., 2021).

DMT activity has demonstrated efficacy in enhancing resilience, as well as mood and relaxation, in chronic pain patients (Shim et al., 2017) and in improving distress and anxiety in cancer patients (Bryl et al., 2024). Incorporating DMT elements into physical exercises has also been effective in enhancing the fitness and overall functioning of older nursing home patients in wheelchairs (Wołoszyn et al., 2021) and improving mood of older people in acute hospital settings (Bungay et al., 2022). Furthermore, it has been effective in enhancing general wellbeing and perceived quality of life of healthy population including the olders (Sebastiani et al., 2026). Despite these findings, to our knowledge, no study has yet evaluated the cardiovascular effects of DMT. To address this critical gap in the literature, the objective of the present study was to verify the potential effectiveness of DMT in improving HRV in a sample of Italian healthy individuals over the age of 65.

Our working hypothesis is grounded, by one hand, on preceding findings demonstrating a correlation between elevated HRV and enhanced physical and mental health and, on the other hand, on evidences about effectiveness of DMT in promoting wellbeing among older people. We then hypothesized that participation in the DMT activity may yield favorable outcomes in terms of enhancing vagal-mediated HRV within the present sample of over-65 individuals. A possible DMT-induced reduction in skin conductance tonic level (SCL) was also expected, since this is considered a marker of tonic sympathetic activity and a useful indicator of stress levels, with higher SCL values generally correlating with higher levels of arousal and stress. Furthermore, since poor sleep quality and sleep deprivation have been found to be associated with reduced HRV (Jackowska et al., 2012) and changes in sleep naturally occur with age (e.g. decreased sleep efficiency and more frequent nocturnal awakenings), a secondary objective of the present study was to verify whether sleep quality could influence the HRV of our sample of older people and whether the DMT activity could modulate not only sleep quality *per se* but also the possible association between HRV and sleep quality.

Finally, since older people are generally at greater risk of depression than younger people (Szymkowicz et al., 2023), often because of their living conditions, and depression severity is negatively associated with HRV, a further aim was to investigate whether the presence of depressive symptoms in our sample of older people could influence HRV and whether the participation in DMT activity could reduce depression symptoms and modulate the possible HRV-depression association.

To address these objectives, we adopted a one-group pretest-posttest approach, a quasi-experimental method commonly used in behavioral and social science research to evaluate the potential effects of an activity on a specific population. The full methodological details are provided in the Methods section.

## 2 Material and methods

### 2.1 Participant recruitment and eligibility criteria

Recruitment advertisements were prepared and disseminated via flyers that were distributed at territorial associations and gyms, and also through local newspaper channels. People who answered the advert were interviewed by PP or SD, study investigators, to collect some general personal information (like age, sex^2^, previous job, general health, and how much exercise they did before and now). This information was put into a database to facilitate selection based on the inclusion/exclusion criteria. Participants of both sex were included in the study if they met the following eligibility criteria: they were over 65 of age; they were not affected by neurological (e.g., Parkinson's disease), psychiatric (e.g. social phobia), musculoskeletal (e.g. arthritis), cardiovascular (e.g. hyperthension) disorders; they were able to carry out their daily activities independently; they reported participating in leisure motor activities to a moderate extent, such as walking at a brisk pace, cycling on flat terrain, gym activities (e.g., pilates), ballroom dance, for approximately one hour, two or three times a week; they have a body mass index (BMI) in the range of 23 − 29 kg/m^2^, which is recommended for people over the age of 65; they do not drink coffee regularly or make moderate use of it (no more than two coffees a day). A total of 27 persons (females, *n* = 17) were found to be eligible and enrolled in the study. The study was performed in accordance with the Declaration of Helsinki ethical standards and approved by the Committee on Bioethics of the University of Pisa (resolution no. 29/2024 of 26/07/2024).

### 2.2 Experimental design and instruments

As anticipated in the Introduction, we here adopted a one-group pretest-posttest approach to assess the potential impact of DMT activity on healthy older adults.

Participants took part in a eight-week DMT cycle on a once-weekly basis. The entire DMT cycle was preceded (pre-DMT) and followed (post-DMT) by a one-hour session where participants answered the self-report questionnaires Geriatric Depression Scale (GDS) and Pittsburg Quality of Sleep Index (PSQI), and resting state physiological parameters (ECG and GSR) were collected. The pre-DMT and post-DMT sessions were done within one week before and after the entire DMT activity, respectively.

#### 2.2.1 DMT protocol

Two DMT cycles were scheduled over the course of two successive periods of the year: Cycle 1 from January to March and Cycle 2 from March to May. Participants were assigned to one cycle or the other in order to obtain two groups (1st and 2nd cycle) of similar size (1st cycle, n=13, and 2nd cycle, n=14). and with a similar female-to-male ratio (1st cycle, 9 F and 4 M; 2nd cycle, 9 F and 5 M). The decision to divide the participants into two groups was taken in accordance with the recommendation of the DMT professional (MR), associated with APID (APID, 2024), who planned the settings and conducted the DMT meetings. This professional indicated that a sample size of 14 would be optimal for facilitating the development of group dynamics. The DMT protocol consisted of eight weekly DMT meetings, each lasting about one hour, which took place in a standard gym facility with sufficient space for unrestrained movement. All the DMT meetings of the cycle were scheduled at the same time of the day, namely from 9:30 to 10:30 a.m. In the second cycle of DMT, the same settings and order as in the first cycle were applied. Each DMT meeting was centered around a specific thematic element (e.g., exploration of the space around the body; finding unusual ways to move). The theme was developed within various phases that typically involved individual, couples, and group motor dialogues, with or without the use of accessories (e.g., elastic ribbons, masks, veils). With very few exceptions, almost all phases were accompanied by appropriate music. A more detailed description of the DMT protocol is enclosed in the Supplementary Material (DMT Protocol).

The DMT professional registered participants' attendance at the DMT cycle at each of the eight DMT meetings. Only those who attended at least six out of eight meetings and did not report any motor disturbances throughout the cycle were included in the subsequent analysis. However, one male participant from the 1st cycle attended only 3 of the 8 DMT sessions and was therefore not included in the subsequent analysis. One male participant from the second cycle was excluded from the analysis due to a minor bicycle accident that impaired his movement during subsequent DMT sessions. None of the other participants reported relevant events outside their daily routine. It is noteworthy that the remaining participants attended a minimum of 85% of the DMT sessions, with no reported instances of motor disturbances throughout the duration of the DMT cycle. Thus, the analyzed sample comprised two groups: 1st cycle, n=12, and 2nd cycle, n=13. Demographic data are presented in Table 1.

**Table 1:** Demographic characteristics of participants.

Cycle	1	2
Sample size	*N* = 12	*N* = 13
**Age (years, mean±SD)**	68.25± 3.41	72.18 ± 6.26
**Sex**	%	%
Female	66.7	72.73
Male	33.3	27.27
**General Health (subjective evaluation)**	N	N
Fairly good	1	4
Good	7	5
Very good	3	4
Excellent	1	0
**Ongoing leisure motor activities**	N	N
Walks	10	11
Cycling	6	7
Dance	1	1
Gym	1	2
Swimming	0	1
**Previous motor activities**	N	N
Yes	11	13
No	1	0

#### 2.2.2 Pre-DMT and post-DMT experimental sessions

The pre-DMT and post-DMT experimental sessions were scheduled between 9:30 a.m and 3:00 p.m. At the beginning of the pre-DMT session, each participant was informed of the experimental procedures and read and signed the informed consent to participate in the study. To ensure comparable conditions between sessions, each participant's post-DMT recording was scheduled at a similar time of day as their pre-DMT session. In addition, participants were instructed to refrain from vigorous physical activity prior to testing and from caffeine consumption for at least two hours before each session. For participants scheduled in the early afternoon, a light meal at lunch was recommended.

#### 2.2.3 ECG and skin conductance recordings

These recordings were carried out during the pre-DMT and post-DMT sessions. The participants were invited to sit in a comfortable armchair and equipped with the (G-Nautilus 32 system, 2026) (gTech, Schiedlberg, Austria) to record synchronized electroencephalogram (EEG) and ECG signals and the wireless wearable sensor (Shimmer3 GSR+, 2026) for galvanic skin response (GSR) recording. The recording session lasted about 13 minutes. For ECG recording, 2 Ag/AgCl disposable electrodes were placed on the chest, according to ECG lead I, and connected to the EEG headset. Signals were acquired by means of the g.Recorder software (sampling frequency 500 Hz, band pass filtering 0.1 − 100 Hz) and ECG was analyzed using Kubios HRV Premium software (Tarvainen et al., 2014) [manual in Kubios (2025)] to obtain the series of consecutive distances between successive R peaks of ECG (RR series) and Heart Rate variability (HRV) measures. For each session, HRV analysis was performed on the entire 13-minute recording interval. Baseline wander and low-frequency non-stationary trends were removed using the smoothness priors detrending algorithm (λ = 250). R-peak detection was visually inspected for each participant, and ectopic beats or artifacts were corrected using Kubios' automatic artifact correction algorithm. The following HRV components in the time and frequency domains were analyzed: the RR intervals (to assess heart rate); the root mean square of the successive differences between RR intervals (RMSSD), primarily reflecting parasympathetic (vagal) nervous system activity; the Stress Index (SI), which is the square root of Baevsky's stress index (Baevsky and Chernikova, 2017) and serves as an indicator of sympathetic activity; the high frequency (HF, 0.15 − 0.4 Hz) components of the power spectrum density of the RR interval series that primarily reflect vagal activity related to the respiratory sinus arrhytmia. We also analyzed a non linear metric derived from Poincaré plots that is SD1, that represents short-term HRV and is related to parasympathetic activity (Tarvainen et al., 2014). The electrodermal activity was monitored using the wireless wearable sensor (Shimmer3 GSR+, 2026) (sampling rate 128 hz), which allows real-time monitoring of the galvanic skin response (GSR). GSR is measured by passing a small current through a pair of electrodes placed on the palmar surface of two fingers of the non dominant hand. The skin conductance level (SCL, microSiemens), which consists of the slowly varying base signal of the GSR (i.e. tonic GSR), was measured as the average of data collected in the same temporal window as the ECG. The ECG-derived and SCL data were subsequently averaged over the 13-minute recording interval. Thereafter, analysis was conducted on the mean values obtained.

#### 2.2.4 Questionnaires

After each the recording session participants were invited to compile the italian version of the Geriatric Depression scale (GDS) (Galeoto et al., 2018) and of the Pittsburgh Sleep Quality questionnaire (PSQI) (Buysse et al., 1989; Curcio et al., 2013).

The GDS is a 30-item instrument designed to evaluate the emerging symptoms of depression in elderly individuals. The scale comprises a series of questions and statements that are devised to evaluate the mood, feelings and behavior of older people. The assessment encompasses the patient's perception of their daily activities, their general mood, their sense of worthlessness, and any symptoms related to memory and cognitive function. Responses are collected in a binary format, with users providing a “Yes” or “No” response to each question. The final score can range from 0 (no symptoms) to 30 (severe depression) and can be interpreted as follows: 0-9, no depression; 10-19, mild depression; 20-30, severe depression.

The PSQI (Pittsburgh Sleep Questionnaire) is a self-report questionnaire frequently implemented in the evaluation of sleep quality. The tool is regarded as valuable due to its multifaceted approach, incorporating both subjective experiences and objective parameters. The global PSQI score (range 0–21) is obtained by summing seven component scores (range 0–3). 3 indicates the greatest degree of dysfunction. Higher scores indicate poorer sleep quality, with scores over 5 suggesting substantial sleep difficulties.

### 2.3 Statistical analysis

#### 2.3.1 Preliminary analysis

The normality of data distribution was evaluated using the Kolmogorov-Smirnov test. As data were found normally distributed, parametric statistical analysis was used.

The ECG recording of two participant, one of Cycle 1 and one of Cycle 2, were excluded from analysis because of some interferences that affected the recording during the Pre-DMT session. Two other subjects in Cycle 2 were excluded because their heart rate was below 50 beats per minute (bradycardia). Thus, HRV analysis was conducted on a total of 21 participants (Cycle 1, *n* = 11; Cycle 2, *n* = 10). Similarly, due to technical problems, the galvanic response of two participants of Cycle 2 could not be adequately recorded. Therefore, SCL analysis was conducted on a total of 23 participants (Cycle 1, *n* = 12; Cycle 2, *n* = 11).

RR and SCL data of the participants of the two cycles were preliminarly compared by means of separate repeated measures ANOVA with DMT (pre-DMT, post-DMT) as within-subject factor and Cycle as between-subject factor (Cycle 1, Cycle 2). Results showed that RR scores of the Cycle 2 (mean ± SD, 948.47 ± 115.34) were significantly [F_(1, 19)_ = 7.15, *p* = 0.015, η^2^ = 0.273; power= 0.72] higher than the RR scores of Cycle 1 (mean ± SD, 813.74 ± 115.34). Nevertheless, a lack of significant DMT × cycle interaction was found [F_(1, 19)_ = 0.154, *p* = 0.699], thereby suggesting that the DMT activity did not exert differential influence upon the cardiac activity of participants of the two cycles.

Results showed that SCL scores from Cycle 2 (0.486 ± 0.285) were not different from those in Cycle 1 (0.513 ± 0.284). In fact, no significant Cycle effect [F_(1, 20)_ = 0.051, *p* = 0.823] nor DMT x Cycle interaction [F_(1, 21)_ = 3.932, *p* = 0.061] were found. Consequently, for the purpose of subsequent analysis, data of participants from the two cycles were aggregated.

#### 2.3.2 Data analysis

The mean scores of the following metrics, GDS, PSQI, HRV parasympathetic indices (RMSSD, HF, SD1), Stress Index (SI), and SCL, measured in the two sessions, were analyzed by means of separated repeated measures analysis of variance (rmANOVA) with DMT (Pre-DMT and Post-DMT) as within-subjects factor, and Age as covariate. These analyses were conducted on the final dataset available for each measure (*N* = 25 for GDS and PSQI scores, *N* = 21 for HRV indices and *N* = 23 for SCL).

The possible association between Age and HRV indices and SCL was studied by means of Pearson correlation analysis. A linear regression model was used to analyse whether GDS and PSQI were predictive of HRV indices. For all statistical test significance was set at *p* < 0.05. To adjust for multiple comparisons across univariate follow-up tests, p-values were corrected using the Benjamini-Hochberg procedure to control the False Discovery Rate (FDR) at *q* < 0.05. To evaluate the statistical adequacy of the sample, a sensitivity power analysis was performed using G*Power (v3.1.9.7) for repeated-measures ANOVA (within-subject factor, 2 time points). Based on the smallest analytical sample (*N* = 21), with α = 0.05, 1−β = 0.80, and a conservative repeated-measures correlation of *r* = 0.65, the design provides adequate power to detect medium-to-large within-subject effects (*f* = 0.31, partial η^2^ = 0.09). The SPSS.15 statistical package was used for all analyses (see SPSS.15, 2006).

## 3 Results

### 3.1 HRV and SCL results

The mean values (± standard deviation) of RR and HRV indices relative to the pre- and post-DMT sessions are reported in Table 2. Repeated measures ANOVA (rmANOVA) analysis conducted on RR and HRV parasympathetic indices in the time (RMSSD) and frequency (HF) domains as well as in the non-linear domain (SD1), with DMT as within-subjects factor and Age as a covariate, yielded a significant overall DMT effect (Multivariate test, *F*(4, 16) = 3.028, *p* = 0.049, partial η^2^ = 0.431, power=0.667). Also, univariate within-subjects analysis revealed significant differences between pre-DMT and post-DMT for RMSSD (F(1, 19)=6.020, p=0.024, partial η^2^ = 0.241, 95% CI [0.97, 5.78], power=0.644), HF (F(1, 19)=9.535, p=0.006, partial η^2^ = 0.334, 95% CI [28.64, 103.45], power=0.834) and SD1 (F(1, 19)=6.242, p=0.022, partial η^2^ = 0.247, 95% CI [0.66, 4.06], power=0.659). Conversely, no differences were found between pre-DMT and post-DMT scores for RR values (F(1,19)=0.039, p=0.846, partial η^2^ = 0.002, 95% CI [-39.10, 57.12], power=0.054).

**Table 2:** RR and HRV-derived parasympathetic and sympathetic indices. In the first column, “pre” and “post” refer to the pre- and post-DMT sessions, respectively.

Session	RR (ms)	RMSSD (ms)	HF (ms^2^)	SD1 (ms)	SI (dimless)
	Mean ±SD	Mean ±SD	Mean ±SD	Mean ±SD	Mean ±SD
pre	873.29 ± 156.51	14.10 ± 5.55	79.71 ± 50.29	10.01 ± 3.94	18.16 ± 7.16
post	882.51 ± 124.91	17.48 ± 7.94	144.76 ± 131.25	12.37 ± 5.61	18.22 ± 6.62

No significant differences (rmANOVA) were found between the pre-DMT and post-DMT scores for the HRV-related sympathetic marker SI (Stress Index) (F(1,19)=1.658,p=0.213, partial η^2^ = 0.080, 95% CI [-3.95, 3.83], power=0.193).

Repeated measures ANOVA conducted on SCL mean scores, with DMT as within-subject factor and Age as a covariate, did not yield any significant difference between the pre-DMT (0.502 ± 0.368) and post-DMT (0.498 ± 0.325) scores (F(1,21)=0.598, p=0.448, partial η^2^ = 0.028, 95% CI [-0.77, 0.18], power=0.114).

### 3.2 GDS and PSQI results

The mean GDS scores of the group in the pre-DMT session indicated an overall absence of depressive symptoms (mean ± SD, 7.68 ± 4.27), with only eight out of 25 participants reporting scores above the cutoff (cutoff = 9) indicative of mild depression (Galeoto et al., 2018). Similar GDS scores were observed subsequent to the DMT (mean ± SD, 7.34 ± 4.80). Indeed, rmANOVA, with DMT as within-subject factor and Age as a covariate, did not yield any significant difference between the pre-DMT and Post-DMT scores (F(1,23)=0.510, p=0.510, partial η^2^ = 0.019, 95% CI [-0.71, 1.39], power=0.098).

The mean PSQI scores of the group in the pre-DMT session (mean ± SD, 5.20 ± 2.83) indicated a negligible deterioration of sleep quality (cutoff = 5) which is consistent with the age-related physiological changes in some sleep parameters (e.g., reduction in total sleep time; increase in night awakenings) (Crowley, 2011). Similar PSQI scores were found in the post-DMT session (5.48 ± 2.93). Indeed, rmANOVA, with DMT as within-subject factor and Age as a covariate, did not yield any significant difference between the pre-DMT and Post-DMT scores (F(1,23)=1.02, p=0.321, partial η^2^ = 0.043, 95% CI [-0.99, 0.44], power=0.163). The above results imply a negligible effect of DMT on both the depression scale and the sleep quality of participants.

### 3.3 Correlation analysis

Pearson correlation analysis between Age and parasympathetic indices revealed weak (*r* < 0.3) non significant positive linear correlations between Age and pre-DMT scores (first three lines of Table 3). However, these correlations became much stronger and significant for the post-DMT scores (*r*>0.54)(last three lines of Table 3). As an example, in Figure 1 the linear associations between Age and the pre- and post-DMT RMSSD (left panel) and SD1 (right panel) scores are shown. A significant increase clearly emerges when comparing pre-DMT and post-DMT associations, and this qualitative observation is quantitatively confirmed by the statistical analyses reported in Table 3. Pearson's correlation analysis also showed a negative correlation between RR and SCL mean scores in both sessions (pre-DMT, *r* = −0.483; post-DMT, *r* = −0.421). However, the RR-SCL association was significant only in the pre-DMT session (pre-DMT, *p* = 0.036; post-DMT, *p* = 0.073).

**Table 3:** Pearson correlation between Age and parasympathetic HRV indices.

Session	Variable	Correlation index (r)
Pre-DMT	RMSSD	0.266
	HF	0.207
	SD1	0.257
Post-DMT	RMSSD	0.578^*^
	HF	0.544^*^
	SD1	0.579^*^

^*^Significance at *p* < 0.01.

**Figure 1:**
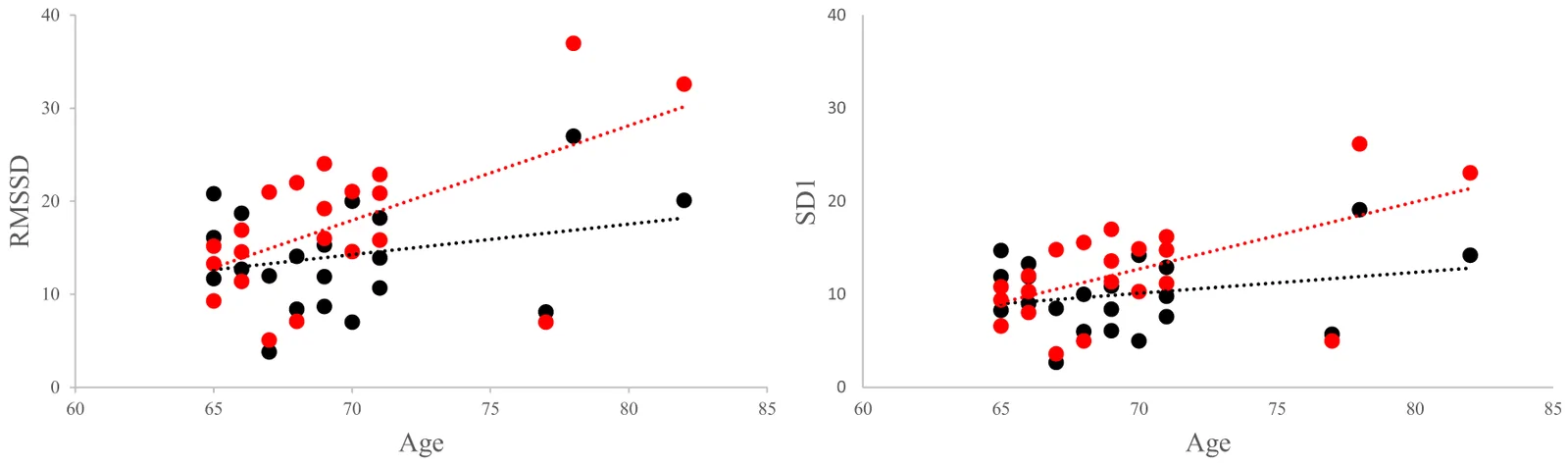
Correlation between age and pre-DMT (black dots) and post-DMT (red dots) scores of RMSSD and SD1 indices. Trendlines are shown.

### 3.4 Regression analysis questionnaires vs. physiological indices

The linear regression model indicated that, in the pre-DMT session, GDS scores were predictive of RR (Figure 2). In fact, the model indicated a good significant overall fit (R-square=0.223; F_(1, 20)_=5.465, *p* = 0.030). The regression equation results to be:


RR=a+b*GDS; a=742.615; b=17.534

thus indicating that, for each one point increase in the GDS score, RR increases by 17.534 percentage points. The linear regression results, including the model summary, analysis of variance (ANOVA) and coefficient tables [evaluated with (SPSS.15, 2006)], are reported in the Supplementary Material.

**Figure 2:**
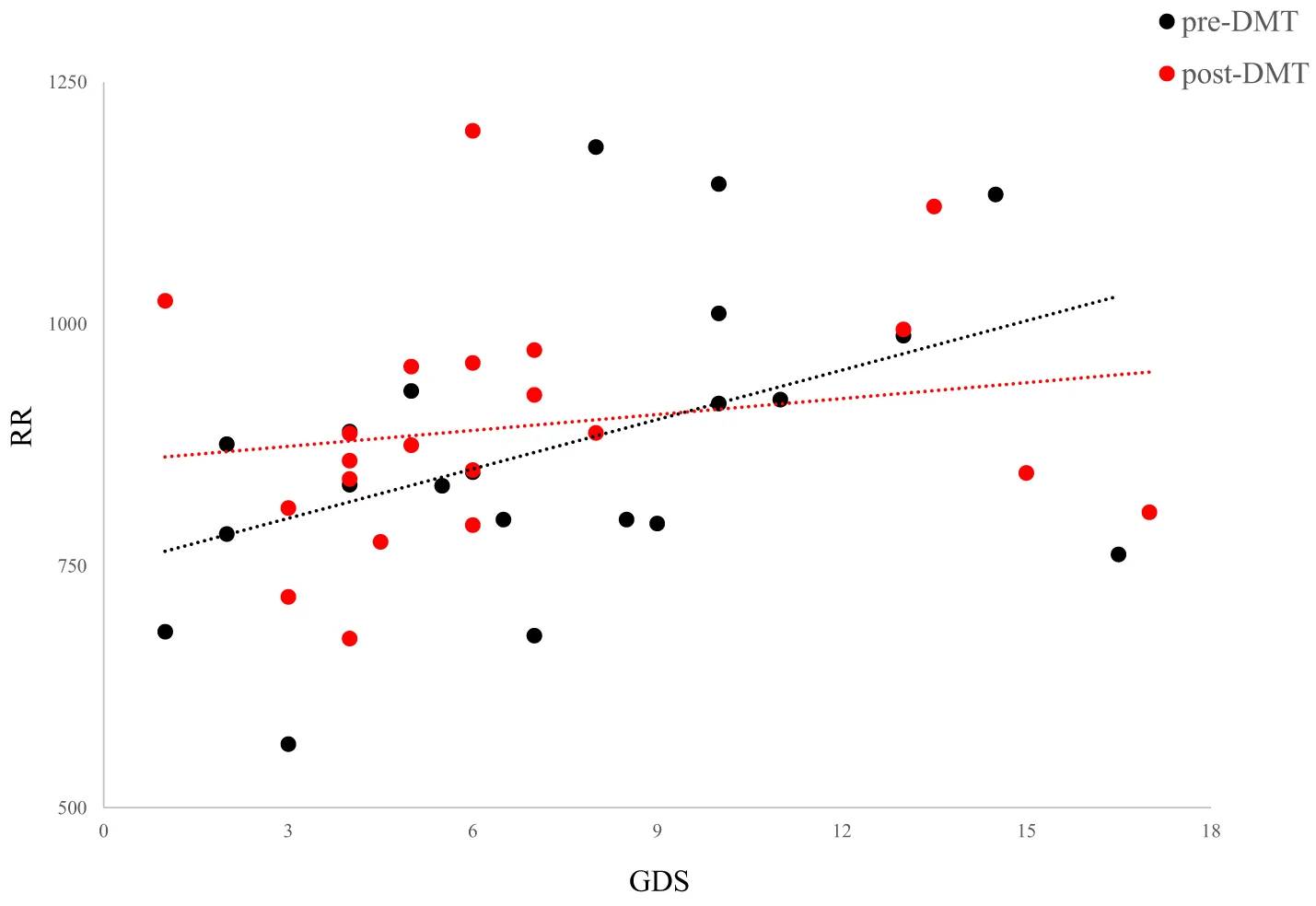
Association between GDS scores and mean RR in the pre-DMT (black dots) and post-DMT (red dots) sessions. Trendlines are shown.

No predictive association was found in the pre-DMT session between GDS scores and HRV indices, GDS scores and SCL as well as between PSQI and RR, HRV indices and SCL.

The association between GDS scores and RR disappeared in the post-DMT session (Figure 2). Predictive associations were still not found in the post-DMT session between GDS scores and HRV indices, GDS scores and SCL as well as between PSQI and all physiological indices (RR, HRV indices and SCL).

## 4 Discussion

The present study is the first to assess the potential positive effects on the cardiovascular system of a dance-based intervention that explicitly integrates body-mind connectivity, emotional expression, and relational dynamics, namely Dance Movement Therapy, in healthy older adults. This extends previous work, which has focused predominantly on technology-based dance training [e.g., exergaming (Varas-Diaz et al., 2020)] or fitness-oriented dance interventions (e.g., Cadore et al., 2014).

The primary finding is that after a 8-week DMT activity a significant increase in resting state HRV parameters (RMSSD, HF, SD1), which are indicators of the parasympathetic outflow to the heart via the vagus nerve, (Pumprla et al., 2002) was found. Previous research has shown that high resting state values of RMSSD, HF and SD1, which are known to be positively correlated with each other (Shaffer and Ginsberg, 2017), indicate an active parasympathetic nervous system. The high parasympathetic activity is closely associated with better down-regulation of negative affect, use of adaptive regulatory strategies, more flexible emotional responding (Balzarotti et al., 2017) and higher cognitive functions [e.g., attention (Thayer and Brosschot, 2005) and motor imagery (Sebastiani et al., 2019)].

This result, that is, the possible efficacy of DMT in improving cardiovascular autonomic regulation in older healthy people, is assessed by post-DMT HRV changes with respect to pre-DMT. This is in agreement with previous studies that employed some form of dance training (i.e., exergaming-based and virtual reality-based dance trainings) as a tool to improve autonomic regulation in the elderly demographic (Sampaio et al., 2016; Varas-Diaz et al., 2020). Nevertheless, the suggested effectiveness of DMT in improving vagally-mediated HRV can not only be attributable to physical activity associated with a pure dance training, but might also be due to the activation of social skills that free body movements stimulate. In fact, the distinctive nature of the DMT approach is the employment of dance and movement to encourage individuals to establish relationships with one another within group dynamics (Dulicai and Hill, 2007; Winters Fisher, 2019; Goodill, 2025; Overby et al., 2025).

Interestingly, in the context of social cognition and behavior, it has been hypothesized that there is a relationship between these domains and vagally-mediated HRV (Geisler et al., 2013; Lischke et al., 2018; Quintana et al., 2012). In fact, HRV has been suggested as a biomarker for adaptive behavior during social interactions. The existence of this relationship is supported by anatomical and functional data. Indeed, changes in the structure and function of the brain regions involved in social cognition and interactions (prefrontal and paralimbic brain areas) are also associated with changes in vagally-mediated HRV (Mulcahy et al., 2019). Also, marked differences in social cognition and interactions have been found between individuals with different vagal-mediated HRV (Beffara et al., 2016). Therefore, it can be hypothesized that the increased vagal activity after the participation in DMT activity may be a manifestation of the improvement in social behavior it facilitated. Clearly, the link between the improved HRV metrics and the relational aspects of DMT is, at this stage, speculative and this aspect would deserve further investigations.

Another interesting finding is the strengthening of the (positive) correlation between age and vagal-related HRV indices. In fact, this association becomes much stronger after DMT, thus indicating both a higher vagal control in the eldest participants but also a role of DMT in reinforcing the age-HRV relationship. The higher vagal control in the eldest people appears to contradict previous findings suggesting a decline in HRV with age (De Meersman and Stein, 2007). However, it is generally accepted that HRV values tend to stabilize after the age of 50 (Umetani et al., 1998). It is noteworthy that the sample of old people under consideration consists of individuals who participate in leisure motor activities to a moderate extent, such as walking and cycling. They also have a normal BMI, are not affected by any mental or physical disorder and have a fairly good sleep quality. Therefore, given that all these factors are involved in the maintenance of optimal HRV, the observation that vagal indices are higher in the eldest participants underscores the notion that, in this demographic, good health and physical and social activity are effective in reducing age-related vulnerabilities to factors such as stress, anxiety and loneliness, that have the potential to compromise cardiac function.

The SCL-RR negative correlation essentially is found not to change between pre- and post-DMT sessions, even if it is worth noting that only during the pre-DMT session the correlation reached a level of statistical significance. This is not an unexpected outcome. In fact, under conditions of rest, the autonomic balance is dominated by the tonic control of the parasympathetic nervous system, which is associated with a reduced sympathetic drive.

A positive causal association is found between GDS and mean RR. This means that individuals who report a higher number of depressive symptoms display lower heart rates. This finding contrasts with previous research indicating an inverse relationship between GDS and HRV indices, including mean RR (Aydın and Kabakçı, 2025; Rouch et al., 2022). However, it is noteworthy that the mean GDS score fell below the cut-off point for depression, with only a few participants reporting scores indicating very mild depression. Therefore, we can hypothesize that the previously reported negative association emerges exclusively in patients clinically diagnosed with depression. However, it is intriguing to note that administration of DMT disrupts the positive association between GDS and mean RR.

Regarding PSQI, in contrast with previous findings indicating that diminished sleep quality is associated with reduced HRV (Jackowska et al., 2012), we find no significant association between PSQI and HRV indices. However, the PSQI scores of both sessions reveal that sleep quality in our sample is relatively good. Only a small number of participants demonstrate scores slightly above the cutoff value, which is consistent with the physiological age-related changes in sleep structure (Crowley, 2011). Therefore, the absence of association between PSQI and HRV and of DMT-related enhancements in the participants' sleep quality is not unexpected, given that their current sleep quality is already satisfactory.

### 4.1 Limitations and conclusion

The results highlighted in this study are encouraging and further validation is required on a larger sample. Indeed, the small sample size represents a key limitation, primarily due to a complex enrollment process: despite a high response rate to recruitment advertisements, very few candidates met the eligibility criteria. However, this small sample also aligns with clinical recommendations, as the DMT professional strongly advised capping group size at 14 participants per cycle to ensure optimal group dynamics. Furthermore, we put forth that, as already discussed in the Introduction, we applied a one-group pretest-posttest design, thus implementing a well-defined and largely exploited quasi-experimental research method (Cranmer, 2017). Further advances in our investigation should involve the use of a control group in order to confirm our preliminary findings. However, this raises questions about which control protocol would be the most appropriate, e.g., structured physical activity, sedentary social activity, or no additional physical or social activity.

However, as a countermeasure regarding the lack of a control group, we paid particular attention to any salient personal events or experiences that participants encountered during the eight-week DMT activity [the so called “history effects” (Cranmer, 2017)], which might have affected the physiological outcome. Indeed, one participant of the Cycle 2, who underwent minor incidents, was excluded from analyses, while none of the other participants reported events outside their daily routine.

Another limitation is the imbalance in the gender of participants, with the majority of the subjects being female. The gender imbalance seen in dance-related activities for older adults has been reported previously, with most studies including a higher percentage of females (Hwang and Braun, 2015). The present study attempted to mitigate this imbalance, maintaining a similar female-to-male ratio in both DMT cycles. One final issue is that the study only examined the short-term effects of DMT on HRV and SCL. Future studies may involve taking repeated measurements over short and long periods of time. In conclusion, the present study provides preliminary evidence that DMT could be an effective strategy for improving the health and wellbeing of older people by preventing or counteracting the age-related decline in HRV that is frequently observed. We argued that this may be ascribed to the DMT peculiarity of stimulating individuals interaction and integration in a collective unit (Goodill, 2025; Overby et al., 2025). In this regard, DMT has the potential to promote a shared positive state of mind and facilitate social interactions and wellbeing, also among most senior individuals (Sebastiani et al., 2026). Finally, based on these premises, the present study may also be relevant to the underlying framework of embodied cognition (Paradisi et al., 2021; Goodill, 2025).

## Data Availability

The raw data supporting the conclusions of this article will be made available by the authors, without undue reservation.

## Footnotes

1 DMT is defined and regulated by the American Dance Therapy Association (ADTA) (ADTA, 2020) and the European Association Dance Movement Therapy (EADMT) (EADMT, 2021). Italian DMT professionals are associated with the Italian Professional Association of Dance-Movement-Therapy [Associazione Professionale Italiana DMT, APID (APID, 2024)], which also provides three-year training courses with a final thesis/dissertation and a practical test.

2 The sex categories female/male were referred to “sex assigned at birth”.

## References

[B1] ADTA (2020). Adta-American Dance Therapy Association. Available online at: https://www.adta.org/ (Accessed January 07, 2026).

[B2] APID (2024). Associazione Professionale Italiana Danzamovimentoterapia. Available online at: https://www.apid.it/ (Accessed January 7, 2026).

[B3] Arakaki X. Arechavala R. J. Choy E. H. Bautista J. Bliss B. Molloy C. . (2023). The connection between heart rate variability (hrv), neurological health, and cognition: A literature review. Front. Neurosci. 17:1055445. doi: 10.3389/fnins.2023.1055445 36937689 PMC10014754

[B4] Archer E. Blair S. N. (2011). Physical activity and the prevention of cardiovascular disease: from evolution to epidemiology. Prog. Cardiovasc. Dis. 53, 387–396. doi: 10.1016/j.pcad.2011.02.006 21545924

[B5] Aydın L. Kabakçı S. (2025). Heart rate variability changes in elderly patients with depression: a systematic review. Psikiyatride Guncel Yaklasimlar - Current Approaches in Psychiatry 17, 716–725. doi: 10.18863/pgy.1556204

[B6] Baevsky R. Chernikova A. (2017). Heart rate variability analysis: physiological foundations and main methods. Cardiometry 2017:66–76. doi: 10.12710/cardiometry.2017.10.6676

[B7] Balzarotti S. Biassoni F. Colombo B. Ciceri M. (2017). Cardiac vagal control as a marker of emotion regulation in healthy adults: a review. Biol. Psychol. 130, 54–66. doi: 10.1016/j.biopsycho.2017.10.008 29079304

[B8] Beffara B. Bret A. Vermeulen N. Mermillod M. (2016). Resting high frequency heart rate variability selectively predicts cooperative behavior. Physiol. Behav. 164, 417–428. doi: 10.1016/j.physbeh.2016.06.011 27343804

[B9] Brown L. Karmakar C. Gray R. Jindal R. Lim T. Bryant C. (2018). Heart rate variability alterations in late life depression: a meta-analysis. J. Affective Disord. 235, 456–466. doi: 10.1016/j.jad.2018.04.071 29679898

[B10] Bryl K. Whitley J. Lopez-Nieves I. Liou K. Chimonas S. Tortora S. . (2024). Experiences and perceived benefits of remotely delivered dance/movement therapy for adult cancer patients: a multi-method program evaluation. Support. Care Cancer 32:6. doi: 10.1007/s00520-024-08591-3 PMC11234514 38802666

[B11] Bungay H. Hughes S. Jacobs C. Zhang J. (2022). Dance for health: the impact of creative dance sessions on older people in an acute hospital setting. Arts & Health 14, 1–13. doi: 10.1080/17533015.2020.1725072 PMC8979553 32028845

[B12] Buysse D. J. Reynolds III C. F. Monk T. H. Berman S. R. Kupfer D. J. (1989). The pittsburgh sleep quality index: a new instrument for psychiatric practice and research. Psychiatry Res. 28, 193–213. doi: 10.1016/0165-1781(89)90047-4 2748771

[B13] Cadore E. L. Pinto R. S. Bottaro M. Izquierdo M. (2014). Strength and endurance training prescription in healthy and frail elderly. Aging Dis. 5, 183–195. doi: 10.14336/ad.2014.0500183 PMC4037310 24900941

[B14] Colzato L. S. Steenbergen L. (2017). High vagally mediated resting-state heart rate variability is associated with superior action cascading. Neuropsychologia 106, 1–6. doi: 10.1016/j.neuropsychologia.2017.08.030 28866318

[B15] Cranmer G. A. (2017). “One-group pretest-posttest design,” in The SAGE Encyclopedia of Communication Research Methods (Thousand Oaks, CA: SAGE Publications, Inc.), 1125–1126.

[B16] Crowley K. (2011). Sleep and sleep disorders in older adults. Neuropsychol. Rev. 21, 41–53. doi: 10.1007/s11065-010-9154-6 21225347

[B17] Curcio G. Tempesta D. Scarlata S. Marzano C. Moroni F. Rossini P. M. . (2013). Validity of the italian version of the pittsburgh sleep quality index (PSQI). Neurol. Sci. 34, 511–519. doi: 10.1007/s10072-012-1085-y 22526760

[B18] de la Parra López V. Panhofer H. (2023). Moments of meeting in DMT: characteristics and implications from the subjective experience of therapists. Am. J. Dance Therapy 45, 41–58. doi: 10.1007/s10465-023-09377-6

[B19] De Meersman R. E. Stein P. K. (2007). Vagal modulation and aging. Biol. Psychol. 74, 165–173. doi: 10.1016/j.biopsycho.2006.04.008 17045727

[B20] Dulicai D. Hill E. S. (2007). “Expressive movement,” in Low-Cost Approaches to Promote Physical and Mental Health: Theory, Research, and Practice, eds. L. L'Abate (New York, NY: Springer New York), 177–200.

[B21] EADMT (2021). The European Association of Dance Movement Therapy (EADMT). Available online at: https://eadmt.com/what-is-dance-movement-therapy-dmt (Accessed January 7, 2026).

[B22] Ernst G. (2017). Heart-rate variability—more than heart beats? Front. Public Health 5:240. doi: 10.3389/fpubh.2017.00240 PMC5600971 28955705

[B23] Esmail A. Vrinceanu T. Lussier M. Predovan D. Berryman N. Houle J. . (2020). Effects of dance/movement training vs. aerobic exercise training on cognition, physical fitness and quality of life in older adults: a randomized controlled trial. J. Bodywork Movement Therap. 24, 212–220. doi: 10.1016/j.jbmt.2019.05.004 31987547

[B24] Galeoto G. Sansoni J. Scucimarri M. Bruni V. De Santis R. Colucci M. . (2018). A psychometric properties evaluation of the italian version of the geriatric depression scale. Depress. Res. Treatm. 2018, 1–7. doi: 10.1155/2018/1797536 PMC5852888 29686898

[B25] Gässler B. Thielmann B. Böckelmann I. Hökelmann A. (2021). Effects of different exercise interventions on heart rate variability and cardiovascular health factors in older adults: a systematic review. Eur. Rev. Aging Phys. Act. 18:24. doi: 10.1186/s11556-021-00278-6 34789148 PMC8597177

[B26] Geisler F. Kubiak T. Siewert K. Weber H. (2013). Cardiac vagal tone is associated with social engagement and self-regulation. Biol. Psychol. 93:13. doi: 10.1016/j.biopsycho.2013.02.013 23466587

[B27] G-Nautilus 32 system (2026). Technical Information About G-Nautilus 32 System. Available online at: https://www.gtec.at/product/g-nautilus-research/ (Accessed January 7, 2026).

[B28] Goodill S. W. (2025). “Dance/movement therapy,” in Undergraduate Research in Dance: A Guide for Students, eds. L. Y. Overby, J. Shanahan, and G. Young (London: Routledge), 103–111.

[B29] Hwang P. Braun K. (2015). The effectiveness of dance interventions to improve older adults' health: a systematic literature review. Alternat. Therap. Health Med. 21, 64–70. PMC5491389 26393993

[B30] Jackowska M. Dockray S. Endrighi R. Hendrickx H. Steptoe A. (2012). Sleep problems and heart rate variability over the working day. J. Sleep Res. 21, 434–440. doi: 10.1111/j.1365-2869.2012.00996.x 22309485

[B31] Kaltsatou A. C. Kouidi E. I. Anifanti M. A. Douka S. I. Deligiannis A. P. (2014). Functional and psychosocial effects of either a traditional dancing or a formal exercising training program in patients with chronic heart failure: a comparative randomized controlled study. Clini. Rehabilitat. 28, 128–138. doi: 10.1177/0269215513492988 23864515

[B32] Kubios (2025). Kubios HRV Scientific User's Guide 2025. Available online at: https://www.kubios.com/downloads/HRV-Scientific-Users-Guide.pdf (Accessed January 7, 2026).

[B33] Laborde S. Mosley E. Thayer J. F. (2017). Heart rate variability and cardiac vagal tone in psychophysiological research – recommendations for experiment planning, data analysis, and data reporting. Front. Psychol. 8:213. doi: 10.3389/fpsyg.2017.00213 PMC5316555 28265249

[B34] Lischke A. Jacksteit R. Mau-Moeller A. Pahnke R. Hamm A. Weippert M. (2018). Heart rate variability is associated with psychosocial stress in distinct social domains. J. Psychosom. Res. 106:5. doi: 10.1016/j.jpsychores.2018.01.005 29455900

[B35] Mulcahy J. S. Larsson D. E. Garfinkel S. N. Critchley H. D. (2019). Heart rate variability as a biomarker in health and affective disorders: a perspective on neuroimaging studies. NeuroImage 202:116072. doi: 10.1016/j.neuroimage.2019.116072 31386920

[B36] Overby L. Shanahan J. Young G. (2025). Undergraduate Research in Dance: A Guide for Students. London: Routledge, 2 edition.

[B37] Paradisi P. Raglianti M. Sebastiani L. (2021). Online communication and body language. Front. Behav. Neurosci. 15:709365. doi: 10.3389/fnbeh.2021.709365 PMC8452979 34557076

[B38] Pumprla J. Howorka K. Groves D. Chester M. Nolan J. (2002). Functional assessment of heart rate variability: physiological basis and practical applications. Int. J. Cardiol. 84, 1–14. doi: 10.1016/S0167-5273(02)00057-8 12104056

[B39] Quintana D. S. Guastella A. J. Outhred T. Hickie I. B. Kemp A. H. (2012). Heart rate variability is associated with emotion recognition: direct evidence for a relationship between the autonomic nervous system and social cognition. Int. J. Psychophysiol. 86, 168–172. doi: 10.1016/j.ijpsycho.2012.08.012 22940643

[B40] Rouch I. Edjolo A. Celle S. Pichot V. Laurent B. Borg C. . (2022). Association between depressive symptoms and long-term heart rate variability in older women: findings from a population-based cohort. J. Affect. Disord. 305, 151–158. doi: 10.1016/j.jad.2022.02.053 35219741

[B41] Sampaio L. Subramaniam S. Arena R. Bhatt T. (2016). Does virtual reality-based kinect dance training paradigm improve autonomic nervous system modulation in individuals with chronic stroke? J. Vascular Intervent. Neurol. 9, 21–29. 27829967 PMC5094257

[B42] Sebastiani L. Daoudagh S. Ignesti G. Raglianti M. Paradisi P. (2026). The impact of dance movement therapy on subjective well-being and quality of life in healthy older adults. Arts and Health 2026:1–18. doi: 10.1080/17533015.2026.2715535 42619251

[B43] Sebastiani L. Di Gruttola F. Incognito O. Menardo E. Santarcangelo E. (2019). The higher the basal vagal tone the better the motor imagery ability. Archives Italiennes de Biologie 157, 3–13. doi: 10.12871/00039829201911 31111952

[B44] Shaffer F. Ginsberg J. P. (2017). An overview of heart rate variability metrics and norms. Front. Public Health 5:258. doi: 10.3389/fpubh.2017.00258 29034226 PMC5624990

[B45] Shim M. Johnson R. B. Gasson S. Goodill S. Jermyn R. Bradt J. (2017). A model of dance/movement therapy for resilience-building in people living with chronic pain. Eur. J. Integrat. Med. 9, 27–40. doi: 10.1016/j.eujim.2017.01.011

[B46] Shimmer3 GSR+ (2026). Technical Information About Shimmer3 GSR+. Available online at: https://www.shimmersensing.com/product/shimmer3-gsr-unit/ (Accessed January 7, 2026).

[B47] Song D. Yu D. Liu T. Wang J. (2024). Effect of an aerobic dancing program on sleep quality for older adults with mild cognitive impairment and poor sleep: a randomized controlled trial. J. Am. Med. Direct. Assoc. 25, 494–499. doi: 10.1016/j.jamda.2023.09.020 39492163

[B48] SPSS.15 (2006). SPSS 15.0 Brief Guide. Available online at: https://users.pop.umn.edu/ruggl001/hist5011/SPSSBriefGuide150.pdf (Accessed January 7, 2026).

[B49] Stenfors C. U. D. Hanson L. M. Theorell T. Osika W. S. (2016). Executive cognitive functioning and cardiovascular autonomic regulation in a population-based sample of working adults. Front. Psychol. 7:1536. doi: 10.3389/fpsyg.2016.01536 27761124 PMC5050226

[B50] Szymkowicz S. M. Gerlach A. R. Homiack D. Taylor W. D. (2023). Biological factors influencing depression in later life: role of aging processes and treatment implications. Translat. Psychiatry 13:1. doi: 10.1038/s41398-023-02464-9 37160884 PMC10169845

[B51] Tan J. P. H. Beilharz J. E. Vollmer-Conna U. Cvejic E. (2019). Heart rate variability as a marker of healthy ageing. Int. J. Cardiol. 275, 101–103. doi: 10.1016/j.ijcard.2018.08.005 30104034

[B52] Tarvainen M. P. Niskanen J.-P. Lipponen J. A. Ranta-aho P. O. Karjalainen P. A. (2014). Kubios hrv - heart rate variability analysis software. Comp. Methods Programs Biomed. 113, 210–220. doi: 10.1016/j.cmpb.2013.07.024 24054542

[B53] Thayer J. F. Brosschot J. F. (2005). Psychosomatics and psychopathology: looking up and down from the brain. Psychoneuroendocrinology 30, 1050–1058. doi: 10.1016/j.psyneuen.2005.04.014 16005156

[B54] Umetani K. Singer D. McCraty R. Atkinson M. (1998). Twenty-four hour time domain heart rate variability and heart rate: Relations to age and gender over nine decades. J. Am. College Cardiol. 31:593–601. doi: 10.1016/S0735-1097(97)00554-8 9502641

[B55] Varas-Diaz G. Subramaniam S. Delgado L. Phillips S. Bhatt T. (2020). Effect of an exergaming-based dance training paradigm on autonomic nervous system modulation in healthy older adults: A randomized controlled trial. J. Aging Physi. Activity 29, 1–9. doi: 10.1123/japa.2019-0452 32702664

[B56] Winters Fisher A. F. (2019). Dance/movement therapy & warrior wellness: a pilot case study. Arts Psychother. 62, 52–60. doi: 10.1016/j.aip.2018.11.010

[B57] Wołoszyn N. Wiśniowska-Szurlej A. Grzegorczyk J. Kwolek A. (2021). The impact of physical exercises with elements of dance movement therapy on the upper limb grip strength and functional performance of elderly wheelchair users living in nursing homes – a randomized control trial. BMC Geriatrics 21:1. doi: 10.1186/s12877-021-02368-7 34247582 PMC8274008

